# Selecting Map Projections in Minimizing Area Distortions in GIS Applications

**DOI:** 10.3390/s8127809

**Published:** 2008-12-03

**Authors:** Faruk Yildirim, Ahmet Kaya

**Affiliations:** Karadeniz Technical University, Engineering Faculty, Dep. of Geodesy and Photogrammetry, 61080, Trabzon, Turkey

**Keywords:** GIS, TM, UTM, Area distortion, Equal-area projection

## Abstract

Various software for Geographical Information Systems (GISs) have been developed and used in many different engineering projects. In GIS applications, map coverage is important in terms of performing reliable and meaningful queries. Map projections can be conformal, equal-area and equidistant. The goal of an application plays an important role in choosing one of those projections. Choosing the equal-area projection for an application in which area information is used (forestry, agriculture, ecosystem etc) reduces the amount of distortion on the area, but many users using GIS ignore this fact and continue to use applications with present map sheets no matter in what map projection it is. For example, extracting area information from data whose country system's map sheet is in conformal projection is relatively more distorted, compared to an equal-area projection one. The goal of this study is to make the best decision in choosing the most proper equal-area projection among the choices provided by ArcGIS 9.0, which is a popular GIS software package, and making a comparison on area errors when conformal projection is used. In this study, the area of parcels chosen in three different regions and geographic coordinates and whose sizes vary between 0.01 to 1,000,000 ha are calculated according to Transversal Mercator (TM, 3°), Universal Transversal Mercator (UTM, 6°) and 14 different equal-area projections existing in the ArcGIS 9.0 GIS software package. The parcel areas calculated with geographical coordinates are accepted as definite. The difference between the sizes calculated according to projection coordinates and real sizes of the parcels are determined. Consequently, the appropriate projections are decided for the areas smaller and equal than 1,000 ha and greater than 1,000 ha in the GIS software package.

## 1. Introduction

The number of GIS applications on large regions has rapidly increased in recent years. The accuracy of data and the correctness of queries used in these applications depend mainly on the choice of map projections constituting the map sheet of the application area. The coordinates on map projections are the transfer of ellipsoidal geographic coordinates onto the map or plane using a proper projection method. Based on these coordinates, some values, such as length, angle, and area are calculated and the required data (statistical information) is acquired. Obtaining such data based on ellipsoidal geographic coordinates using a GIS software package is not possible. It is only possible with map projection coordinates [1, 2].

Area distortion is minimized when the maps are produced with equal-area projection. The level of distortion can be controlled but cannot be removed completely. The area distortion is more in conformal maps (TM, UTM) compared to equal-area maps. Therefore, the transformation to equal-area projection is performed in applications in which area data is important [3-5].

The most correct solution of area calculation for large regions is the one based on ellipsoidal geographic coordinates. However, there is no tool offered in all GIS software packages for area calculations based on these coordinates because of its difficulty. Instead of ellipsoidal geographic coordinates, the calculation is performed based on projection coordinates. This type of calculation does not give the definite size of area but it includes area distortions related to the chosen projection type. Consequently, there is undoubtedly an area distortion in these area calculations. Equal-area projection should be preferred in order to minimize the error. There are different kinds of equal-area projections, as can be seen in projection tools of GIS software. This study aims to help users to make a correct decision about which projection should be chosen or which projection provides minimum distortion in area information [6-8].

For this aim, the equal-area projections available in ArcGIS 9.0 are investigated. Area distortions are evaluated by calculating the areas of parcels, chosen in different districts and different sizes, based on both geographical and equal-area projection coordinates. Furthermore, since TM and UTM are used as map sheets in many countries, the conformal projections (TM, UTM) are compared with the equal-area projections.

### 1.1. Area calculation based on ellipsoidal geographic coordinates

In order to determine the area distortions in projections, the real size of area calculated based on ellipsoidal geographic coordinates should be known. The real value of area that will be calculated on ellipsoid will be used for comparison with the area calculated by using equal-area projection coordinates.

There exist four remarkable methods proposed in the literature by Kimerling [9], Danielsen [10], Gillissen [11] and Sjöberg [12] for calculation of area of a closed figure using ellipsoidal geographic coordinates. Since the Kimerling method is a spherical solution, the borders are not the geodesic curve but the great circle. Therefore, it is not a definite ellipsoidal solution. In the Danielsen and Sjöberg solutions, the parcel borders are taken as a geodesic curve and the area below that curve is calculated as ellipsoidal area. In the Gillissen method the area is calculated based on Albers equal-area projection by dividing part of big circle to the chords. Since the Danielsen and Sjöberg methods depend on series expansion formula, its effect is decreased while the area is growing. On the other hand, the Gillissen method is not a series expansion formula; however, it requires more complex and long operation steps. Considering the calculation methods and tools available today, this hardness can be neglected. This method gives more sensitive results compared to the others while the area is growing, but it is disadvantageous in terms of processing time. Consequently, the Gillissen method was selected in this study to calculate the real sizes of parcels.

### 1.2. Equal-area projections in ArcGIS 9.0 software package

Among the various GIS software available, ArcGIS 9.0 version was used in this study. This version is more convenient and rich in terms of projection choices compared to the old versions (e.g. ArcInfo 8.3). Fourteen equal-area projections available in ArcGIS 9.0 were investigated in the study. TM and UTM projection coordinates were also calculated, except for equal-area projections. The list of the projections and the starting coordinates used in the application are given in Table 1. Note that *B_0_* represents central latitude, *L_0_* stands for central longitude, *B_1_* and *B_2_* represent standard parallels for conic projection, and *R_0_* is mean radius of curvature. The principals and equations of these projections and more are available in many different references. Detailed information can also be found in manuals and help menus of the related software [5, 13-15].

## 2. Application

**Figure 1.**
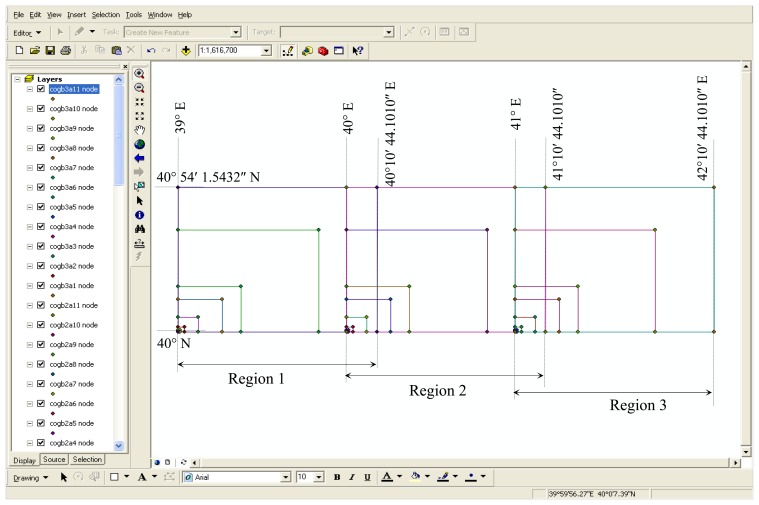
Application parcels.

In order to choose the equal-area projection which has the lowest distortion, the 33 test areas (Figure 1) are formed and their ellipsoidal geographic coordinates are given in Table 2. The lower and upper latitudes of the parcels are common but not the longitudes. There different region are determined by selecting different starting longitudes. The starting longitudes determining the regions are *39°*, *40°* and *41°* respectively. Consequently, the distance between the regions is *1°*. There are 11 parcels in each region and the lower corners of all parcels are chosen as common. The real areas of the parcels whose corners are represented with ellipsoidal geographic coordinates are calculated according to the Gillissen method.

The application has two main goals. First, the chosen areas are calculated based on equal-area and conformal projections and compared with the real area values. Therefore, area distortions of the chosen projections for different size of parcels are studied. In this distortion calculation, only the errors derived from projection choice are investigated, but errors caused by processes such as coordinate production and scale factor are not investigated. Secondly, the effects of distance from starting longitude on area distortions are evaluated with parcels chosen in different regions.

In the application stage, 33 parcels defined by ellipsoidal geographic coordinates are first transformed into the 16 projections given in Table 1. ED50 datum and Hayford International Ellipsoid are used in this transformation. The differences from the real areas are taken by calculating the areas of parcels whose projection corner coordinates are certain. These differences can be seen in Table 3 in m^2^. Since it is selected as an example, only differences for the first region are given in Table 3. The differences for the 2^nd^ and 3^rd^ regions are found to be the same as the 1^st^ region. However, the differences in these regions for the conformal projections are not equal. Besides, it is seen in the Table 3 that distortion is increased when the area grows.

Having compared the conformal projections, it is seen that the distortions in parcels defined by TM coordinates are much smaller compared to the UTM system. This is due to the effect of m_0_ scale factor on coordinates in UTM system. Area distortion is smaller than 1 m^2^ in parcels up to 1,000 ha in size in TM system. Area distortion of a 1,000 ha of parcel which is 1° distant from longitude is 2,000 m^2^ for TM system and 6,000 m^2^ for UTM system. The area distortion is increased in TM projections while the distance to longitude is increased, but it is decreased in UTM projections because of the scale factor.

When the results of equal-area projections are compared, it is seen that the area distortion of Albers, Behrmann, Bonne and Sinusoidal projections are smaller than 1 m^2^ for the parcels up to 1,000 ha. Area distortions for the same projections up to 50,000 ha are smaller than 539 m^2^, 211 m^2^, 1,024 m^2^ and 301 m^2^, respectively. It is seen that Behrmann and Sinusoidal projections give better results as the parcel sizes grow.

## 3. Results

In case of TM or UTM, TM coordinates should be used due to scale factor for precise area calculation. It is because of the fact that m_0_ scale factor in UTM system affects the area distortion. The difference between the areas calculated based on UTM and TM coordinates is in the amount of the square of the scale factor. This should be taken into account while using UTM coordinates in ArcGIS. When the area is calculated using TM coordinates, the difference from real value is smaller than 1 m^2^ for the parcels up to 1,000 ha in regions close to the starting longitude (1^st^ region). This difference is getting larger in conformal projection when the distance to the starting longitude is increased.

When area distortions in equal-area projections are compared, it is seen that Albers, Behrmann, Bonne and Sinusoidal projections (when these projections are independent from the starting longitude) can be used when parcels are up to the 1000 ha in size, and if 1 m^2^ precision is enough. Behrman and Sinusoidal projections could also be suggested up 50,000 ha by preserving the area difference under 500 m^2^. In case of preferring one of these two projections, Behrmann projection can be preferred since it gives more precise results. Unlike conformal projections, getting away from the longitude has no effect on area distortion in these suggested equal-area projections. Projection should be chosen carefully in GIS applications when area information is important. The errors stemming from the location and the scale factor are ignored in this study. The accuracy further decreases when the effects of errors in coordinates resulted from field surveying are considered in large area application in GIS.

## Figures and Tables

**Table 1.** The projection used in the application and start of the coordinates.

**Projection**	***L****_0_*	***B****_0_*	***B****_1_*	***B****_2_*	***R****_0_****=(M****_0_****N****_0_****)****^(1/2)^*
TM	39°,42°				
UTM	39°				
Albers Equal-Area Conic	39°	40 °	40 °	42°	
Equal-Area Cylindirical	39°	40 °			6,374,618.375 m
Behrmann Equal-Area Conic	39°				
Bonne Equal-Area	39°				
Craster Equal-Area Parabolic	39°				
Eckert II Equal Area	39°				
Eckert IV Equal-Area	39°				
Eckert VI Equal-Area	39°				
Hammer-Aitoff Equal-Area	39°				
Lambert Azimutal Equal Area-Conic	39°	40 °			6,374,618.375 m
McBryde-Thomas Flat-Polar Quartic	39°				
Mollweide Equal-Area	39°				
Quartic Authalic Equal-Area	39°				
Sinusoidal Equal-Area	39°				

**Table 2.** The corner points of test parcels.

Parcel No	Corner No	Region 1						Region 2			Region 3			Elipsoidal area (real area)		
		*B*			*L*			*L*			*L*			m^2^	Km^2^	ha
P1	1	40 °	0′	0.00000″	39 °	0′	0.00000″	40 °	0′	0.00000″	41°	0′	0.00000″	**100**	**0.0001**	**0.01**
	2	40 °	0′	0.32421″	39 °	0′	0.00000″	40 °	0′	0.00000″	41°	0′	0.00000″			
	3	40 °	0′	0.32421″	39 °	0′	0.42156″	40 °	0′	0.42156″	41°	0′	0.42156″			
	4	40 °	0′	0.00000″	39 °	0′	0.42156″	40 °	0′	0.42156″	41°	0′	0.42156″			
P2	1	40 °	0′	0.00000″	39 °	0′	0.00000″	40 °	0′	0.00000″	41°	0′	0.00000″	**1000**	**0.001**	**0.1**
	2	40 °	0′	1.02525″	39 °	0′	0.00000″	40 °	0′	0.00000″	41°	0′	0.00000″			
	3	40 °	0′	1.02525″	39 °	0′	1.33308″	40 °	0′	1.33308″	41°	0′	1.33308″			
	4	40 °	0′	0.00000″	39 °	0′	1.33308″	40 °	0′	1.33308″	41°	0′	1.33308″			
P3	1	40 °	0′	0.00000″	39 °	0′	0.00000″	40 °	0′	0.00000″	41°	0′	0.00000″	**10000**	**0.01**	**1**
	2	40 °	0′	3.24214″	39 °	0′	0.00000″	40 °	0′	0.00000″	41°	0′	0.00000″			
	3	40 °	0′	3.24214″	39 °	0′	4.21560″	40 °	0′	4.21560″	41°	0′	4.21560″			
	4	40 °	0′	0.00000″	39 °	0′	4.21560″	40 °	0′	4.21560″	41°	0′	4.21560″			
P4	1	40 °	0′	0.00000″	39 °	0′	0.00000″	40 °	0′	0.00000″	41°	0′	0.00000″	**100000**	**0.1**	**10**
	2	40 °	0′	10.25260″	39 °	0′	0.00000″	40 °	0′	0.00000″	41°	0′	0.00000″			
	3	40 °	0′	10.25260″	39 °	0′	13.33101″	40 °	0′	13.33101″	41°	0′	13.33101″			
	4	40 °	0′	0.00000″	39 °	0′	13.33101″	40 °	0′	13.33101″	41°	0′	13.33101″			
P5	1	40 °	0′	0.00000″	39 °	0′	0.00000″	40 °	0′	0.00000″	41°	0′	0.00000″	**1000000**	**1**	**100**
	2	40 °	0′	32.42137″	39 °	0′	0.00000″	40 °	0′	0.00000″	41°	0′	0.00000″			
	3	40 °	0′	32.42137″	39 °	0′	42.15846″	40 °	0′	42.15846″	41°	0′	42.15846″			
	4	40 °	0′	0.00000″	39 °	0′	42.15846″	40 °	0′	42.15846″	41°	0′	42.15846″			
P6	1	40 °	0′	0.00000″	39 °	0′	0.00000″	40 °	0′	0.00000″	41°	0′	0.00000″	**10000000**	**10**	**1000**
	2	40 °	1′	42.52519″	39 °	0′	0.00000″	40 °	0′	0.00000″	41°	0′	0.00000″			
	3	40 °	1′	42.52519″	39 °	2′	13.33572″	40 °	2′	13.33572″	41°	2′	13.33572″			
	4	40 °	0′	0.00000″	39 °	2′	13.33572″	40 °	2′	13.33572″	41°	2′	13.33572″			
P7	1	40 °	0′	0.00000″	39 °	0′	0.00000″	40 °	0′	0.00000″	41°	0′	0.00000″	**100000000**	**100**	**10000**
	2	40 °	5′	24.21106″	39 °	0′	0.00000″	40 °	0′	0.00000″	41°	0′	0.00000″			
	3	40 °	5′	24.21106″	39 °	7′	1.83466″	40 °	7′	1.83466″	41°	7′	1.83466″						
	4	40 °	0′	0.00000″	39 °	7′	1.83466″	40 °	7′	1.83466″	41°	7′	1.83466″			
P8	1	40 °	0′	0.00000″	39 °	0′	0.00000″	40 °	0′	0.00000″	41°	0′	0.00000″	**500000000**	**500**	**50000**
	2	40 °	12'	4.94793″	39 °	0′	0.00000″	40 °	0′	0.00000″	41°	0′	0.00000″			
	3	40 °	12'	4.94793″	39 °	15'	44.02376″	40 °	15'	44.02376″	41°	15'	44.02376″			
	4	40 °	0′	0.00000″	39 °	15'	44.02376″	40 °	15'	44.02376″	41°	15'	44.02376″			
P9	1	40 °	0′	0.00000″	39 °	0′	0.00000″	40 °	0′	0.00000″	41°	0′	0.00000″	**1000000000**	**1000**	**100000**
	2	40 °	17'	5.21839″	39 °	0′	0.00000″	40 °	0′	0.00000″	41°	0′	0.00000″			
	3	40 °	17'	5.21839″	39 °	22'	15.87541″	40 °	22'	15.87541″	41°	22'	15.87541″			
	4	40 °	0′	0.00000″	39 °	22'	15.87541″	40 °	22'	15.87541″	41°	22'	15.87541″			
P10	1	40 °	0′	0.00000″	39 °	0′	0.00000″	40 °	0′	0.00000″	41°	0′	0.00000″	**5000000000**	**5000**	**500000**
	2	40 °	38'	12.29124″	39 °	0′	0.00000″	40 °	0′	0.00000″	41°	0′	0.00000″			
	3	40 °	38'	1229124″	39 °	49'	54.99890″	40 °	49'	54.99890″	41°	49'	54.99890″			
	4	40 °	0′	0.00000″	39 °	49'	54.99890″	40 °	49'	54.99890″	41°	49'	54.99890″			
P11	1	40 °	0′	0.00000″	39 °	0′	0.00000″	40 °	0′	0.00000″	41°	0′	0.00000″	**10000000000**	**10000**	**1000000**
	2	40 °	54'	1.54315″	39 °	0′	0.00000″	40 °	0′	0.00000″	41°	0′	0.00000″			
	3	40 °	54'	1.54315″	40 °	10'	44.10100 ″	41 °	10'	44.10100 ″	42°	10'	44.10100″			
	4	40 °	0′	0.00000″	40 °	10'	44.10100 ″	41°	10'	44.10100 ″	42°	10'	44.10100″			

**Table 3.** The differences between real areas and projection areas (m^2^).

**Region**	**Parcel**	**ha**	**Albers**	**Behrmann**	**Bonne**	**Craster**	**Cylindir**	**EckertII**
B1	P1	0.01	0.000	0.000	0.000	0.118	-0.114	0.118
	P2	0.1	0.000	0.000	0.000	1.183	-1.136	1.183
	P3	1	0.000	0.000	0.000	11.829	-11.360	11.829
	P4	10	0.000	0.000	0.000	118.272	-113.619	118.272
	P5	100	-0.002	0.001	-0.004	1182.002	-1136.904	1182.004
	P6	1000	-0.217	0.083	-0.410	11797.304	-11391.508	11797.524
	P7	10000	-21.646	8.349	-41.001	117246.383	-114620.258	117268.429
	P8	50000	-539.520	211.566	-1024.586	579504.688	-579372.737	580056.141
	P9	100000	-2153.203	854.850	-4097.036	1148663.740	-1167965.904	1150870.440
	P10	500000	-53314.618	22284.492	-102285.458	5513059.892	-6025430.170	5568320.692
	P11	1000000	-211709.134	91907.001	-408711.559	10657085.601	-12308502.250	10878401.301
**Region**	**Parcel**	**ha**	**EckertIV**	**EckertVI**	**Hammer**	**Lambert**	**McBryde**	**Mollweide**
B1	P1	0.01	0.118	0.118	0.118	0.000	0.118	0.118
	P2	0.1	1.183	1.183	1.183	0.000	1.183	1.183
	P3	1	11.829	11.829	11.829	-0.001	11.829	11.829
	P4	10	118.272	118.272	118.272	-0.033	118.272	118.272
	P5	100	1182.003	1182.003	1182.002	-1.047	1182.002	1182.002
	P6	1000	11797.399	11797.408	11797.299	-33.307	11797.327	11797.323
	P7	10000	117255.903	117256.832	117245.881	-1074.310	117248.712	117248.254
	P8	50000	579742.582	579766.627	579491.653	-12435.734	579562.687	579551.047
	P9	100000	1149615.040	1149713.640	1148610.140	-36071.927	1148895.060	1148847.960
	P10	500000	5536812.882	5539534.902	5511565.642	-445527.358	5518772.092	5517536.412
	P11	1000000	10752003.901	10763666.901	10650640.401	-1349174.054	10679719.901	10674599.601
**Region**	**Parcel**	**ha**	**Quartic**	**Sinusoidal**	**UTM**	**TM**		
B1	P1	0.01	0.118	0.000	-0.080	0.000		
	P2	0.1	1.183	0.000	-0.800	0.000		
	P3	1	11.829	0.000	-7.998	0.000		
	P4	10	118.272	0.000	-79.984	0.000		
	P5	100	1182.002	-0.001	-799.834	0.006		
	P6	1000	11797.296	-0.122	-7997.784	0.617		
	P7	10000	117245.553	-12.159	-79922.510	61.539		
	P8	50000	579483.594	-301.102	-398383.098	1538.132		
	P9	100000	1148578.320	-1195.782	-793692.508	6152.413		
	P10	500000	5510814.002	-28975.148	-3845499.656	153823.378		
	P11	1000000	10647768.401	-113104.959	-7383504.514	615387.698		
